# AI-assessed sarcopenia as an independent predictor of neoadjuvant chemotherapy outcomes in muscle-invasive bladder cancer

**DOI:** 10.1007/s11547-026-02203-2

**Published:** 2026-03-31

**Authors:** Antonella Borrelli, Simone Novelli, Emanuele Messina, Ludovica Laschena, Ailin Dehghanpour, Francesca Mezzapesa, Nicholas Landini, Giuseppe Simone, Maurizio Muscaritoli, Valeria Panebianco

**Affiliations:** 1 Department of Radiological Sciences, Oncology and Pathology, Sapienza University, Sapienza/Policlinico Umberto I, Viale Regina Elena 324, 00161 Rome, Italy; 2 Department of Mechanical and Aerospace Engineering, Sapienza University of Rome, Rome, Italy; 3 Liver Failure Group, Institute for Liver and Digestive Health, UCL Medical School, Royal Free Hospital, London, UK; 4 Department of Urology, IRCCS “Regina Elena” National Cancer Institute, Rome, Italy; 5 Department of Translational and Precision Medicine, Sapienza University of Rome, 00161 Rome, Italy

**Keywords:** Artificial intelligence, Sarcopenia, Bladder cancer, Computed tomography, Magnetic resonance, Oncology

## Abstract

**Purpose:**

Sarcopenia has already been widely investigated as a potential indicator of negative outcomes in oncology patients. Our aim was to evaluate the potential predictive role of sarcopenia assessed using an Artificial Intelligence-powered software in response to neoadjuvant chemotherapy (NAC) in patients with muscle-invasive bladder cancer (MIBC).

**Materials and methods:**

In this single-centre retrospective study, we enrolled patients diagnosed with non-metastatic MIBC who underwent NAC and had available pre-treatment mpMRI of the bladder and baseline CT scan. The follow-up MRI assessment was performed using the NacVI-RADS algorithm to evaluate response to systematic therapy. AI-based software automatically calculated the skeletal muscle index (SMI) from CT images at the L3 vertebral level. Multivariate logistic regression analysis was performed to assess independent predictors of response to NAC, and a receiver operating characteristic (ROC) analysis was subsequently conducted to provide an additional level of statistical validation.

**Results:**

Fifty-five patients were included (mean age: 67.2 years). Sarcopenia was identified in 36.4% of patients. Multivariate logistic regression revealed sarcopenia (OR: 9.08; 95% CI 1.32–61.92; *p* = 0.024), comorbidities (OR: 14.63; 95% CI 2.12–100.71; *p* = 0.006), and high NacVI-RADS scores (4–5) (OR = 2.13 95% CI 1.03–4.42; *p* = 0.042) as independent predictors of poor response to NAC. Receiver operating characteristic (ROC) curve analysis confirmed the high discriminative ability of SMI for predicting treatment response (AUC = 0.952).

**Conclusion:**

Sarcopenia, assessed by AI-powered analysis, was negatively associated with tumor response following NAC in patients with MIBC. These findings support the integration of AI-driven sarcopenia evaluation into clinical staging workflows, enabling tailored nutritional interventions and improved patient stratification. Moreover, our study reinforces the prognostic value of the NacVI-RADS scoring system in predicting NAC outcomes.

**Supplementary Information:**

The online version contains supplementary material available at 10.1007/s11547-026-02203-2.

## Introduction

Sarcopenia, defined as the progressive loss of skeletal muscle mass and strength [1], has emerged as a crucial factor in cancer prognosis and patient outcomes, particularly in uro-oncology.

Studies have demonstrated that sarcopenia is associated with increased morbidity and mortality in bladder cancer patients [2], especially those undergoing major surgical interventions [3, 4]or chemotherapy [5]. Given its impact [6] on treatment response and overall survival, accurate assessment of sarcopenia is essential for risk stratification and therapeutic treatment planning. Muscle-invasive bladder cancer (MIBC) is a significant global health concern requiring a multimodal treatment approach that typically includes radical cystectomy and pelvic lymphadenectomy often preceded by neoadjuvant chemotherapy (NAC) [7].

NAC, recommended by major clinical guidelines [8], aims to improve oncological outcomes by reducing the risk of metastasis. However, its widespread adoption remains limited due to the absence of reliable biomarkers for predicting treatment response.

To address this unmet need, the Neoadjuvant Chemotherapy Vesical Imaging Reporting and Data System (NacVI-RADS) [9] was developed as an extension of the Vesical Imaging Reporting and Data System (VI-RADS) [10]. This mpMRI-based scoring system provides a standardised approach for evaluating bladder cancer response to NAC, aiding clinical decision-making. Despite its utility, additional biomarkers are needed to further refine patient selection and optimize therapeutic strategies.

Sarcopenia holds significant potential as a predictive biomarker in this setting. However, traditional methods for diagnosing sarcopenia, such as dual-energy X-ray absorptiometry (DEXA), Magnetic resonance (MRI) and computed tomography (CT), are often time-consuming, costly, and subject to inter-observer variability. Recent advances in artificial intelligence (AI) have enabled automated, rapid, and reproducible body composition analysis, offering a feasible solution for the objective assessment of sarcopenia [11].

Machine learning and deep learning algorithms can provide objective and standardized evaluations of sarcopenia, making it a feasible and valuable predictive marker in clinical practice [12].

By integrating AI-driven sarcopenia assessment into bladder cancer management clinicians could enhance patient stratification, personalize treatment strategies, and potentially improve oncological outcomes.

The primary objective of this study was to investigate the prognostic value of AI-assessed sarcopenia in patients with muscle-invasive bladder cancer (MIBC) undergoing neoadjuvant chemotherapy (NAC). To this end, we assessed both the radiological response, using the NacVI-RADS classification, and the pathological outcome, defined by the Tumor Regression Grade (TRG). The rationale for this dual approach was that NacVI-RADS provides a non-invasive, preoperative assessment of treatment response, whereas TRG represents the definitive histopathological reference standard. The primary analysis focused on the association between pre-treatment Skeletal Muscle Index (SMI) and pathological response, while secondary analyses explored the relationship between other clinical variables including the post-treatment radiological response evaluated by NacVI-RADS and treatment outcome.

## Materials and methods

### Study design, patient population, and standards of reference

This retrospective, single-center observational study received formal approval from the Institutional Review Board, with a waiver of informed consent. It was conducted in adherence to the guidelines of good clinical practice and the ethical principles outlined in the most recent version of the Declaration of Helsinki [13].

From May 2020 to September 2024, 59 patients with non-metastatic muscle-invasive urothelial bladder cancer (MIBC), diagnosed via transurethral resection of bladder tumor (TURBT) and/or re-resection (Re-TURBT), were referred for neoadjuvant chemotherapy (NAC) followed by radical cystectomy (RC) and eventually extended pelvic lymph node dissection (ePLND).

All participants underwent multiparametric MRI (mpMRI) using a 3 Tesla scanner (Siemens Healthineers, MAGNETOM Vida; GE Healthcare, Discovery 750) for the assessment of VI-RADS and NacVI-RADS score) and computed tomography (CT) scan (Somatom Definition, Siemens Medical Solutions, Forchheim, Germany) both 2–4 weeks prior to staging TURBT and after completing the last NAC cycle.

The clinical and demographic data collected included gender, age, height, weight, smoking, presence of hydronephrosis, hematuria, tumor multifocality, and comorbidity. Comorbidities were defined as the presence of at least one clinically relevant chronic disease (e.g., cardiovascular disease, chronic respiratory disease, diabetes mellitus, or chronic renal insufficiency), as documented in patients’ medical records. Chronic inflammatory pathology in active status referred to any ongoing inflammatory disease (e.g., inflammatory bowel disease, rheumatoid arthritis, or chronic hepatitis) showing clinical and/or laboratory evidence of active inflammation at the time of inclusion.

Inclusion criteria were: age > 18 years, histologically confirmed diagnosis of urothelial tumor, availability of baseline CT scans and MRI before and after NAC, and eligibility for cisplatin-based chemotherapy. Patients with a life expectancy lower than 3 months or affected by any chronic inflammatory pathology in active status, with no long-term clinical information available, unsuitable for chemotherapy treatment, were excluded. Patients unable to complete the full course of NAC were excluded, as well as cases with poor MRI image quality precluding reliable assessment or absence of abdominal CT scans for sarcopenia evaluation. The process of patient selection, inclusion, and exclusion is illustrated in the STROBE flowchart (Supplementary Fig. 1).

The NacVI-RADS score was evaluated in comparison to final histopathological findings and tumor regression grade (TRG) derived from radical cistectomy specimens. TURBT and RC pathological samples were analyzed by an experienced uro-pathologist with 15 years of experience in the field. The AI-powered software was applied to the first CT images to calculate the Skeletal Muscle Index (SMI-L3) in order to assess sarcopenia.

### Imaging acquisition and analysis

All patients underwent baseline multiparametric MRI (mpMRI) of the bladder and contrast-enhanced CT of the abdomen and pelvis before starting NAC. For this study, only the baseline CT scans were considered for AI-based sarcopenia assessment, while mpMRI was repeated after NAC to evaluate treatment response according to the NacVI-RADS system.

All imaging studies included in this analysis, were acquired as part of routine clinical care. No additional imaging was conducted specifically for research purposes.

### MRI protocol

Both pre-treatment and post-treatment MRI scans were performed following the acquisition protocol recommended by the original VI-RADS document, using the same scanner and parameters whenever possible to ensure methodological consistency. The majority of examinations were acquired on 3-Tesla scanners (Siemens Healthineers MAGNETOM Vida; GE Healthcare Discovery 750) equipped with a 32-channel phased-array body coil. A 1.5-Tesla scanner (Siemens Healthineers Avanto) was used only in patients with contraindications to 3T MRI (e.g., presence of cardiac devices).

Patients, when not contraindicated, received an intramuscular antispasmodic agent (n-butyl-scopolamine 20 mg) to reduce bladder wall motion and were requested to drink 500–1000 mL of water 30 min before the examination to achieve proper bladder distension [14]. The highest b-value used to acquire DWI sequences was 1000 s/mm^2^. After precontrast imaging, a gadolinium-based contrast agent was administered using a power injector at a dose of 0.1 mmol/kg of body weight and an injection rate of 3.0 mL/s for standard relaxivity agents, followed by a saline flush [10].

Image analysis was performed independently by two experienced radiologists with 10 and 4 years of experience in bladder imaging.

For each patient, at the primary MRI, the lesion with the highest VI-RADS score was considered as the index. A VI-RADS cut-off score of ≥ 3 to define MIBC was assumed.

### CT protocol

All CT images before neoadjuvant chemotherapy were acquired using multidetector CT scanners (Somatom Sensation 64 or Somatom Definition; Siemens Healthineers, Erlangen, Germany) with standardized acquisition parameters for all patients. These CT scans, routinely performed as part of the standard staging protocol for urothelial tumors prior to initiating neoadjuvant therapy, were utilized for body composition analysis.

Scanning parameters were as follows: tube voltage 120 kVp; tube current 100–250 mAs; pitch 1.2; and collimation 0.625–0.75 mm. Images were reconstructed using a 1-mm slice thickness on axial, coronal, and sagittal planes, applying both soft tissue kernel (B31f) and lung kernel (B75f) reconstruction.

The CT acquisition protocol adhered to standard CT urography practice and included the following phases: an unenhanced phase; a corticomedullary phase acquired approximately 35–45 s after intravenous injection of a non-ionic iodinated contrast medium; a nephrographic phase (80–100 s); and an excretory phase (10–15 min). An iodinated non-ionic contrast agent was administered intravenously at a dose calculated according to patient body weight (approximately 1.2–1.7 mL/kg), in line with institutional CT urography protocols. The exact volume varied depending on the specific contrast medium used.

### AI imaging analysis

**Fig. 1:**
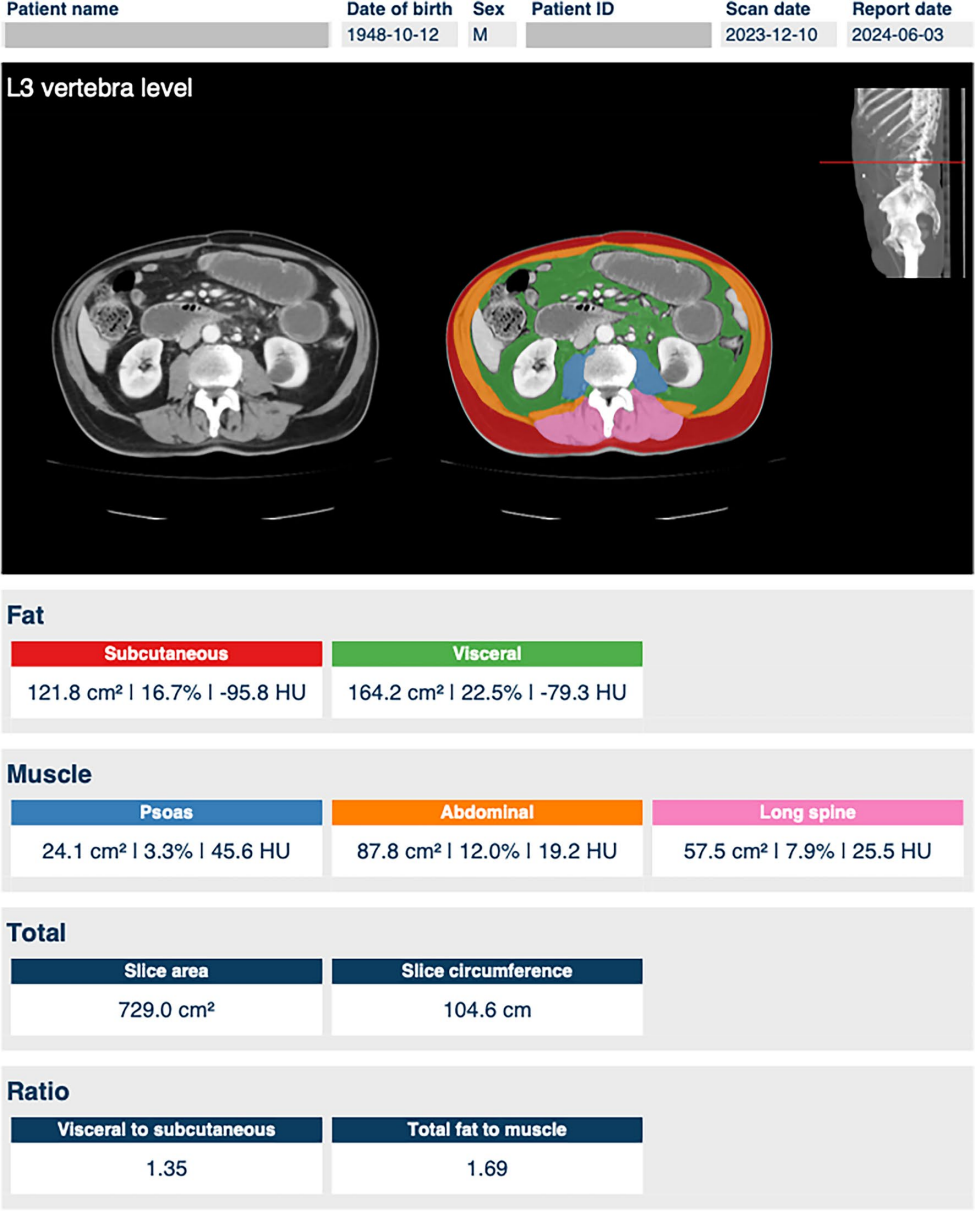
Case example of the automatic segmentation performed by the software at the third lumbar vertebra level in a 76-year-old male patient diagnosed with MIBC

The Quantib body composition® software (Rotterdam, Netherlands) was used to measure body composition quantitatively. This software analysed CT images of patients taken during CT examinations stored in our institutional picture archiving and communication system (PACS), selecting just the non-contrast phase. The software focused on the L3 vertebral body level and automatically segmented the images to determine the areas of the abdominal, psoas, and long spine muscles. Finally, the software generated a form displaying the relevant values in a few minutes, as shown in Fig. 1.

Patients in whom the software produced grossly incorrect segmentations (i.e., severe artefacts, failure to adequately identify skeletal muscle structures at the L3 level, or complete mislabelling of muscle regions) were excluded from the analysis. Minor imperfections were tolerated, while manual corrections were not performed since the software does not allow for manual editing of segmentations. All analyses were performed locally on a workstation directly connected to the institutional PACS, without any transfer or upload of patient data to external or cloud-based servers.

### Chemotherapy regimen

In line with the European Association of Urology (EAU) guidelines, patients received 3–4 cycles of cisplatin-based combination chemotherapy (cisplatin plus gemcitabine) prior to cystectomy. The regimen consisted of gemcitabine 1,000 mg/m^2^ administered intravenously on days 1 and 8, and cisplatin 70 mg/m^2^ administered intravenously on day 1 of each 21-day cycle, provided creatinine clearance was ≥ 60 mL/min. Antiemetics and hydration protocols were administered according to institutional standards. Creatinine clearance was calculated for all patients to ensure cisplatin eligibility with a minimum requirement of CCr ≥ 60 mL/min. The chemotherapy regimen was delivered every 21 days. With drug administration on both Day 1 and Day 8. On Day 1, Gemcitabine was infused over 30 min in 250–500 mL of sodium chloride 0.9%. Cisplatin was administered over 60 min in 500 mL of sodium chloride 0.9%. Following the hydration protocol. On Day 8, Gemcitabine infusion was repeated over 30 min in 250–500 mL of sodium chloride 0.9%. Antiemetics were administered as pre-medication according to institutional guidelines.

### AI-based sarcopenia and response to therapy assessment

Based on CT-AI-powered software, the SMA (Skeletal Muscle Area) was obtained by summing the muscle areas of the psoas, abdominal, and long spine muscles.

SMI (Skeletal Muscle Index) was obtained by normalising the SMA by the patients’ squared height and represents a more appropriate tool to define sarcopenia than the psoas muscle volume. According to the literature, we defined sarcopenia as SMI < 55 cm^2^/m^2^ for men and SMI < 39 cm^2^/m^2^ for women. However, no universally standardized thresholds for the diagnosis of sarcopenia currently exist, particularly in oncology patients and in those affected by urogenital tumors. Therefore, we adopted the cut-off values suggested in previous studies [15, 16].

The assessment of the follow-up MRI after the NAC cycles was performed using a NacVI-RADS algorithm for evaluation of response to therapy (Neoadjuvant Chemotherapy VI-RADS—nacVI-RADS) [17].

NacVI-RADS categorically defines complete Radiological Response (RaR) based on prior VI-RADS score, presence of residual disease, tumor size, and infiltration of the muscularis propria.

Complete RaR was labeled in case of the absence of intravesical lesions after NAC completion (NacVI-RADS 1–2). Partial RaR was defined as any radiological downstaging with evidence of residual disease, with or without evidence of muscle invasiveness (NacVI-RADS 3–4).

**Fig. 2:**
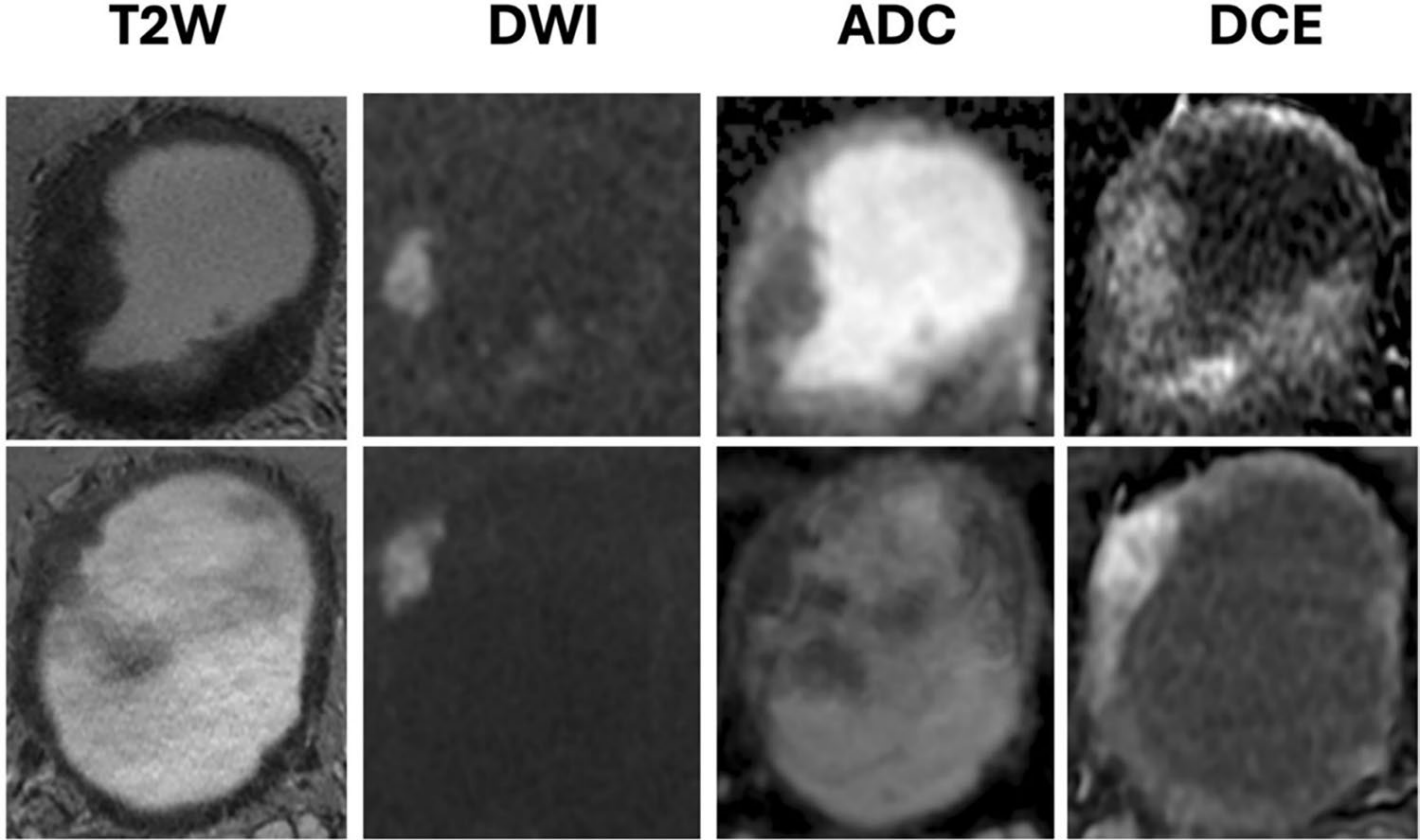
Case example of a VI-RADS 4 in a Sarcopenic 67 years old male patient (SMI value = 41.43 cm^2^/m^2^) with a sessile lesion located on the right lateral bladder wall. After neoadjuvant chemotherapy, the lesion still infiltrates the muscularis propria layer. Showing no downstaging and no measurable reduction in size. Therefore, it indicates a NacVI-RADS score of 5. T2W: T2-weighted imaging; DWI: diffusion-weighted imaging; ADC: apparent diffusion coefficient); DCE: dynamic contrast enhancement)

No RaR was defined as lack of radiological downstaging and/or upstaging regardless prior VI-RADS scoring (NacVI-RADS 5) (Fig. 2).

### Statistical analysis

All statistical analyses were performed using the Statistical Package for the Social Sciences (SPSS) version 28. All tests were two-sided, and statistical significance was set at *p* < 0.05.

The primary outcome variable was non-response to neoadjuvant chemotherapy, defined as pathological TRG 3 at radical cystectomy. Accordingly, higher odds ratios (OR > 1) indicate a greater likelihood of non-response. Univariable and multivariable analyses were performed to evaluate associations between clinicopathological and radiological features, including age (70 < vs. ≥ 70), smoking history (no vs yes), and presence of hematuria (absence vs micro- vs macrohematuria). number of foci pre-NAC (1 vs. > 1), presence of hydronephrosis (absence vs presence), the primary VI-RADS (3–4 vs. 5), post-treatment NacVI-RADS (both 1–2-3 vs. 4–5 and 1–2 vs. 3–4–5) and TRG (1–2 vs. 3).

To assess the discriminative performance of the skeletal muscle index (SMI) in predicting pathological response, a Receiver Operating Characteristic (ROC) curve analysis was performed. The area under the curve (AUC) and 95% confidence interval (CI) were calculated.

## Results

### Patient characteristics

Overall, 59 patients were enrolled. Three patients had to interrupt the treatment after the second administration of cisplatin due to nephrotoxicity (eGFR < 30 mL/min) and were therefore excluded from the analysis. In addition, one patient was excluded because of a segmentation error on the baseline CT scan, resulting in a final study population of 55 patients, 46 males (84%) and 9 females (16%), with a median age of 69 (interquartile range (IQR) 66–72 years).

The demographic, radiologic and clinical characteristics of the cohort are summarised in Table 1.

**Table 1:** Summary of cohort population’s clinical, radiological and pathological data

jlm	Responder n (%) (TRG 1–2)	Non-Responder n (%) (TRG 3)	*p*-value
	Yes	No	
*Age n (%)*			
< 70	13 (23.64%)	19 (34.55%)	*p* = 0.87
> 70	8 (14.55%)	15 (27.27%)	
*Smoking. n (%)*			
Yes	7 (12.73%)	22 (40%)	*p* = 0.04
No	14 (25.45%)	12 (21.82%)	
*Gender n (%)*			
Male	5 (9.09%)	3 (5.45%)	*p* = 0.12
Female	16 (29.09%)	31 (56.36%)	
*Hydronephrosis n (%)*			
Yes	0 (0%)	2 (3.64%)	*p* = 0.69
No	34 (61.82%)	19 (34.55%)	
*Hematuria n (%)*			*p* = 0.35
Yes	16 (29.09%)	24 (43.64%)	
No	4 (7.27%)	11 (20%)	
*Multifocality n (%)*			
Yes	2 (3.64%)	5 (9.09%)	*p* = 0.54
No	19 (34.55)	28 (50.91%)	
*Comorbidity n (%)*			
Yes	10 (18.18%)	19 (34.55%)	*p* = 0.34
No	11 (20.00%)	15 (27.27%)	
*VIRADS n (%)*			
3–4	29 (52.73%)	28 (50.91%)	*p* = 0.91
5	26 (47.27%)	27 (49.09%)	
*NacVIRADS n (%)*			
1	10 (58.82%)	7 (41.18%)	p = 0.04
2	7 (53.84%)	6 (46.16%)	
3	4 (57.14%)	3 (42.86%)	
4	5(83.33%)	1 (16.67%)	*p* = 0.03
5	8 (66.67%)	4 (33.33%)	
*Sarcopenia n (%)*			
Yes	5 (9.09%)	25 (45.45%)	*p* = 0.003
No	16 (29.09%)	9 (16.36%)	

	Median	IQR	
SMA responder	135.2	(120.1–201.4)	*p* = 0.068
SMA non responder	98.7	(72.5–156.8)	
SMI responder	47.3	(37.04–77.65)	*p* = 0.048
SMI non responder	38.6	(32.86–96.95)	

### Sarcopenia and pathological response

A striking difference was noted in sarcopenia prevalence. Among responders, only 9.09% exhibited sarcopenia, whereas 45.45% of non-responders had sarcopenia, indicating a highly significant association (*p* = 0.003). Although chemotherapy responders exhibited a higher median skeletal muscle area (SMA) compared to non-responders [135.7 cm^2^ (IQR: 120.1–201.4) vs 98.7 cm^2^ (IQR: 72.5–156.8)], this difference did not reach statistical significance (*p* = 0.068). Conversely, the median SMI was also significantly greater in responders (47.3 vs. 38.6. *p* < 0.01), suggesting a strong association between higher muscle mass and improved treatment response.

Baseline clinical and radiological characteristics stratified by sarcopenic status are summarized in Table 2.

**Table 2:** Baseline clinical and radiological characteristics stratified by sarcopenic status

Variable	Total (*n* = 55)	Sarcopenic (*n* = 20)	Non-sarcopenic (*n* = 35)	*p*-value
Age, years (median, IQR)	69 (66–72)	71 (68–74)	68 (65–70)	0.04*
Gender (male), *n* (%)	46 (84%)	15 (75%)	31 (89%)	0.21
Gender (female), *n* (%)	9 (16%)	5 (25%)	4 (11%)	–
BMI, kg/m^2^ (mean ± SD)	25.4 ± 3.1	23.8 ± 2.7	26.3 ± 3.0	0.01*
ECOG (PS ≥ 1), *n* (%)	19 (35%)	11 (55%)	8 (23%)	0.03*
Comorbidities (≥ 2), *n* (%)	17 (31%)	10 (50%)	7 (20%)	0.02*
Smoking n (%)	38 (69%)	14 (70%)	24 (69%)	0.95
Hydronephrosis *n* (%)	12 (22%)	6 (30%)	6 (17%)	0.31
VI-RADS ≥ 5, n (%)	28 (51%)	12 (60%)	16 (46%)	0.29
nacVI-RADS 4–5, *n* (%)	20 (36%)	13 (65%)	7 (20%)	0.002*
TRG 3 (non-responder), *n* (%)	22 (40%)	12 (60%)	10 (29%)	0.01*

No significant difference was observed in sex distribution between groups.

Patients with sarcopenia were significantly older, had lower BMI values, a higher comorbidity burden, and worse ECOG performance status compared with non-sarcopenic patients. Sarcopenic patients also showed higher post-treatment NacVI-RADS scores and a greater proportion of pathological non-responders (TRG 3).

### Radiological response (NacVI-RADS) and clinical predictors

Univariate logistic regression analysis assessed the relationship between clinicopathological and radiological features and treatment response (Table 3).

**Table 3:** Univariable and multivariable regression analysis assessing the correlation between clinicopathological and radiological features and response to systemic treatment

Sample size	*p* value*	Odds ratio	*p* value**	Odds ratio
Multifocality	*p* = 0.13	5.18 (0.59–45.57)		
Smoking	*p* = 0.193	0.46 (0.133–1.504)		
Gender	*p* = 0.083	1.16 (0.220–4.862)		
Hydronephrosis	*p* = 0.2	2.31 (0.63 -8.477)		
Hematuria	*p* = 0.25	2 (0.612–6.540)		
Comorbidity	***p* = 0.00**	16.66 (3.94–70.36)	***p* = 0.006**	14.627 (2.12–100.71)
VIRADS	*p* = 0.83	1.61 (0.629–4.28)		
NacVI-RADS 1–2-3 4–5	***p* < 0.001**	36.6 (4.36–307.92)	***p* = 0.042**	2.13 (1.03–4.42)
NacVI-RADS 1–2 3–4-5	***p* = 0.03**	1.88 (1.08–24.94)	*p* = 0.502	1.15 (0.25–5.29)
Sarcopenia n (%)	***p* = 0.01**	8.89 (2.52–31.35)	***p* = 0.024**	9.08 (1.32–61.92)

Statistically significant *p*-values are shown in bold

Several variables were found to have statistically significant associations with treatment outcomes.

One of the most notable findings was the strong association between comorbidity and poor treatment response.

In the univariate analysis, several variables were assessed for their association with the outcome of interest. Comorbidity demonstrated a significant association (*p* < 0.001) with an odds ratio (OR) of 16.66 (95% Confidence Interval [CI] 3.94–70.36).

To explore the clinical behaviour of NacVI-RADS category 3, two grouping strategies were evaluated: 1–2 vs. 3–4–5 and 1–2–3 vs. 4–5.

In the univariable analysis, both classifications were significantly associated with treatment outcome. Specifically, higher NacVI-RADS categories (4–5) were strongly associated with non-response (p < 0.001; OR = 36.6; 95% CI 4.36–307.92), while the 1–2 vs. 3–4–5 grouping also reached significance (*p* = 0.03; OR = 1.88; 95% CI 1.08–24.94). Sarcopenia was another strong predictor of poor treatment response (*p* = 0.01; OR = 8.89; 95% CI 2.52–31.35).

In the multivariable analysis, comorbidity remained significantly associated with non-response (*p* = 0.006; OR = 14.63; 95% CI 2.12–100.71) and NacVI-RADS 1–2–3 vs. 4–5 retained statistical significance (*p* = 0.042; OR = 2.13 (95% CI 1.03–4.42). Conversely, the 1–2 vs. 3–4–5 grouping did not retain significance (*p* = 0.502; OR = 1.15; 95% CI 0.25–5.29) suggesting that category 3 behaves more similarly to the non–muscle-invasive group. Sarcopenia also remained an independent predictor of poor response (*p* = 0.024; OR = 9.08; 95% CI 1.32–61.92). The relatively wide confidence intervals observed for some variables likely reflect the limited sample size Other variables did not show significant associations in the multivariable model.

### Predictive performance: ROC analysis

**Fig. 3:**
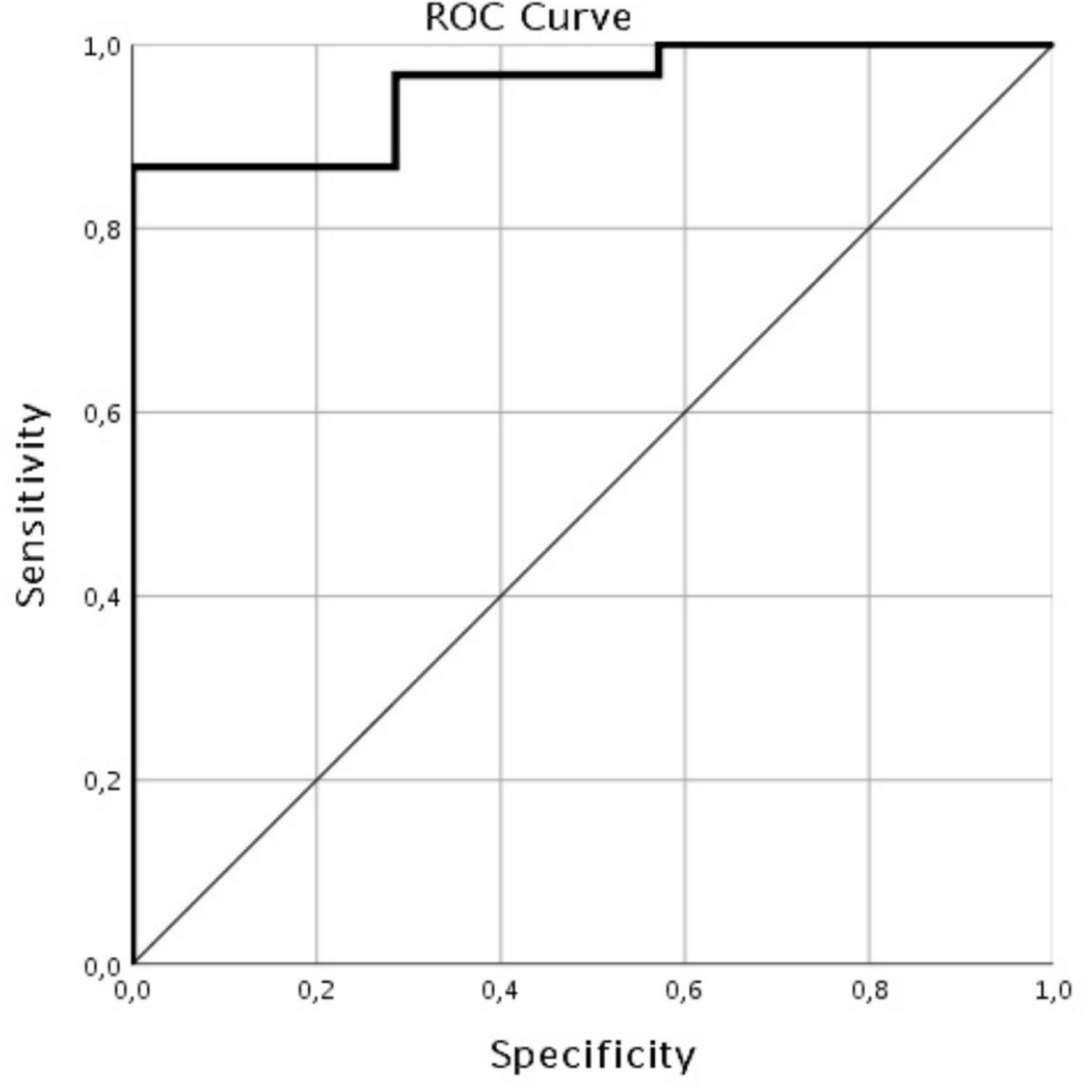
Receiver operating characteristic (ROC) curve illustrating the diagnostic performance of skeletal muscle index (SMI) in predicting tumor regression grade (TRG)

To complement the logistic regression analysis and provide a more comprehensive evaluation of the predictive performance of sarcopenia, we conducted a Receiver Operating Characteristic (ROC) curve analysis using the Skeletal Muscle Index (SMI) as a continuous variable. The resulting area under the curve (AUC) was 0.952 (95% CI 0.89–0.99), indicating excellent discriminative ability of SMI in distinguishing between responders (TRG 1–2) and non-responders (TRG 3) to neoadjuvant chemotherapy (Fig. 3). This analysis was performed to evaluate the predictive performance of SMI as a continuous variable rather than to determine a new threshold. Although the high AUC reflects strong separation between the two groups, it should be interpreted with caution given the relatively small sample size.

This remarkably high AUC value underscores the strength of SMI as a predictive marker and highlights its potential utility in clinical practice. Notably, the use of SMI as a continuous variable rather than a binary classification based on sarcopenia cutoffs alone enables a more refined risk stratification and may capture clinically meaningful gradients in treatment response likelihood. This finding supports incorporating quantitative body composition metrics into predictive models and clinical decision-making frameworks.

## Discussion

This study aimed to identify clinicopathological and radiological factors predicting treatment response, focusing on applying AI-driven sarcopenia assessment as a predictive tool for response to neoadjuvant therapy in muscle-invasive bladder cancer. Several studies already evaluated the impact of sarcopenia status in muscle-invasive bladder cancer patients after neoadjuvant chemotherapy [18]. However, to our knowledge, no study has yet explored sarcopenia in this context using artificial intelligence-based software. Our primary goal was to validate the prognostic impact of sarcopenia by assessing it through AI-powered CT analysis, specifically to determine its ability to predict objective clinical benefit based on tumor response to systemic neoadjuvant therapy. As expected, one of the most significant findings was the strong association between sarcopenia and poor treatment response. Indeed, the multivariate analysis indicated sarcopenia as an independent factor correlating with non-response to NAC (*p* = 0.024) with an adjusted OR of 9.08 (95% CI 1.32–61.92. *p* = 0.024).

The association between sarcopenia and poor response to chemotherapy may be explained by several biological and clinical mechanisms. Loss of skeletal muscle mass and function can alter drug pharmacokinetics, resulting in reduced systemic exposure to cytotoxic agents and consequently lower therapeutic efficacy [19, 20]. Furthermore, sarcopenia is associated with chronic low-grade inflammation, insulin resistance [21], and impaired protein synthesis, which together may create a pro-tumoral microenvironment and reduce cellular resilience to treatment [22]. In addition, sarcopenic patients often present with multiple comorbidities and decreased metabolic reserve, which may limit chemotherapy tolerance and contribute to treatment discontinuation. In our study, sarcopenia remained an independent predictor of non-response even after adjusting for comorbidity, indicating that it exerts an effect beyond general frailty and comorbid burden.

The ROC analysis, performed using tumor regression grade (TRG) 1–2 versus TRG 3 as the reference standard, confirmed the strong discriminative ability of SMI for predicting pathological response (AUC = 0.952; 95% CI 0.89–0.99). This finding supports the role of quantitative body composition metrics as promising imaging biomarkers in the neoadjuvant setting. However, the relatively small sample size and retrospective design may have contributed to an overestimation of the AUC value, and these results should therefore be interpreted with caution and validated in larger, prospective cohorts. SMI proves to be not only a statistically significant factor but also a clinically actionable tool with high prognostic accuracy. These findings highlight the pivotal role played by sarcopenia in predicting treatment outcomes, aligning with previous studies that have linked sarcopenia to adverse treatment responses in urothelial cancer patients, particularly in those undergoing chemotherapy or radiotherapy [15, 16, 23].

The use of AI to assess sarcopenia offers an objective and reproducible measure, providing a valuable tool for identifying at-risk patients who may benefit from more tailored treatment approaches [24].

AI-assisted imaging techniques have shown promise in accurately assessing muscle mass and quality, which are key determinants of sarcopenia. These results are consistent with previous studies that have investigated the role of sarcopenia, assessed using AI, in different stages of the disease [25].

By incorporating AI-based assessments of sarcopenia, clinicians can gain a more precise understanding of a patient's overall physical condition which is critical for making informed treatment decisions.

AI-based workflow can overcome the limitations of manual muscle measurements and provide consistent and reliable results that may improve patient outcomes.

The results of both univariate and multivariate logistic regression analyses revealed several other key findings that highlight the importance of Nac-VIRADS scores in predicting treatment outcomes.

Our results emphasise the prognostic relevance of Nac-VIRADS classification, particularly when stratifying patients using the 1–2–3 vs. 4–5 cutoff. This approach was found to be more meaningful compared to the 1–2 vs. 3–4–5 classification, as category 3 likely represents cases that are no longer muscle-invasive rather than those with persistent or worsening disease. The inclusion of NacVI-RADS category 3 within the non-muscle-invasive group aligns with emerging clinical evidence suggesting that these cases do not exhibit the same aggressive characteristics as categories 4 and 5 [17].

The statistical significance of Nac-VIRADS (1–2–3 vs. 4–5) in both univariate and multivariate analyses reinforces its value as an independent predictor of poor treatment response and adverse prognosis. Conversely, the alternative cut-off (1–2 vs. 3–4-5) did not retain significance in multivariate analysis. Probably because category 3 includes cases that have responded to treatment and are no longer muscle-invasive. These findings suggest that revising the current classification framework to reflect category 3 as an indicator of response could enhance its clinical utility and improve risk stratification.

Overall, this study highlights the importance of sarcopenia as a predictive factor for treatment response particularly when assessed using AI-based techniques.

The use of AI to quantify sarcopenia may offer valuable insights into the patient's ability to tolerate treatment and to predict outcomes more accurately than traditional methods. Given the growing evidence supporting the prognostic value of sarcopenia incorporating AI-based assessments into clinical practice could significantly improve personalized treatment strategies, ultimately leading to better patient management and outcomes.

Future studies should further explore the integration of AI in assessing sarcopenia and other predictive factors as well as the development of tailored interventions for patients at high risk of poor treatment response.

## Limitations

While the present study provides important insights into potential predictors of treatment response, it is not without limitations. First, its retrospective design and the single-centre setting may introduce bias and limit the validation of the findings.

Second, the relatively small sample size, although comparable to other studies in this field, could reduce the statistical power of the analysis and limit the ability to detect subtle difference between groups. In addition, the AI tool used in our analysis does not allow manual correction of segmentation errors, a limitation that further restricted the dataset and contributed to the relatively small sample size.

Furthermore, the exclusion of patients who did not complete neoadjuvant chemotherapy or had non-analyzable CT scans may have introduced a selection bias toward fitter individuals, potentially influencing the observed associations.

We focused exclusively on the SMI score; however, nutritional status can also be evaluated through biological markers (e.g. albumin levels, neutrophil-to-lymphocyte ratio, Nutrition Risk Index) or functional indicators (e.g. physical performance assessments, peak expiratory flow). Comparing and incorporating these additional markers could provide a more comprehensive understanding of the multidimensional nature of nutritional status [26].

Both internal and external validation were not performed, which represents an additional limitation of the present study.

Moreover, it will be interesting to evaluate the impact on survival after a longer follow-up. Finally, our results need to be confirmed by prospective and larger series before they impact the current management of sarcopenic patients to try to optimize outcomes in this group of fragile patients.

## Conclusion

In conclusion, this study highlights the significant role of sarcopenia measured using advanced AI-driven techniques as a robust and independent predictor of response to neoadjuvant therapy. The findings have strong clinical implications underscoring the potential for AI-assisted sarcopenia assessment to guide personalized treatment strategies ultimately improving patient outcomes. Integrating AI-driven sarcopenia assessment into pretreatment workflow could significantly refine therapeutic decision-making in MIBC patients.

## Supplementary Information

Below is the link to the electronic supplementary material.

Supplementary file1 (DOCX 23 kb)
