# Supplementary material for: AI-assessed sarcopenia as an independent predictor of neoadjuvant chemotherapy outcomes in muscle-invasive bladder cancer
Source: Radiol Med. 2026 Mar 31;131(7):1217–27. doi: 10.1007/s11547-026-02203-2 (PMC13369673; doi:10.1007/s11547-026-02203-2)
Supplement: Supplementary file 1 — Supplementary file1 (DOCX 23 kb) [file 11547_2026_2203_MOESM1_ESM.docx]

**Supplementary Figure S1.** STROBE flowchart showing 59 patients enrolled (4 excluded) with a final cohort of 55 patients.

Patients with MIBC histologically confirmed (n =59)

Final cohort (n=55)

Patient excluded

- Unsuitable for chemotherapy treatment (n=3)
- AI segmentation error (n=1)
